# Effects of intravenous glucose and lipids on innate immune cell activation in healthy, obese, and type 2 diabetic subjects

**DOI:** 10.14814/phy2.12249

**Published:** 2015-02-12

**Authors:** Peter Horvath, Stacy R Oliver, Frank P Zaldivar, Shlomit Radom-Aizik, Pietro R Galassetti

**Affiliations:** 1 Institute for Clinical Translational Science, University of CaliforniaIrvine, California; 2 Department of Pharmacology, School of Medicine, University of CaliforniaIrvine, California; 3 Pediatric Exercise Research Center, University of CaliforniaIrvine, California

**Keywords:** Glucose, granulocytes, lipids, monocytes

## Abstract

Atherosclerosis/cardiovascular disease are major causes of morbidity/mortality in obesity and type 2 diabetes (T2D), and have been associated with activation of innate immune cells, their diapedesis to the arterial intima and formation of the atherosclerotic plaque. While in obesity/T2D immune cell activation likely depends on dysregulated metabolism, the interaction between individual metabolic factors typical of these conditions (hyperglycemia, hyperlipidemia), innate immune cell activation, and the progression of atherosclerosis remains unclear. We, therefore, measured by flow cytometry cell surface expression of CD11b, CD14, CD16, CD62L, and CD66b, known markers of granulocyte (Gc) and monocyte (Mc) activation, in five healthy, five obese, and five T2D subjects, during 4-h i.v. infusions of 20% dextrose (raising blood sugar levels to ∼220 mg/dL), 20% Intralipid (raising trygliceride levels to ∼6 mmol/L), or a combination of the two. We hypothesized that both glucose and lipids would increase Gc/Mc surface marker expression, and simultaneous infusion would have an additive or synergistic effect. Surprisingly, though, infusion of glucose alone had little effect, while lipids, alone or combined with glucose, significantly increased expression of several markers (such as CD11b in Gc and Mc, and CD66 b in GC) within 60–90 min. Less pronounced increases in systemic inflammatory cytokines also occurred in obese and T2D subject, with no acute changes in gene expression of the the proinflammatory genes NFκB and CCR2. Our results suggest that lipids may be stronger acute contributors to innate cell activation than acute hyperglycemia per se, possibly helping shape more effective preventive dietary guidelines in T2D.

## Introduction

Atherosclerosis is closely associated with chronic inflammation (Libby 2002), which is a characteristic feature of obesity and type 2 diabetes (T2DM). In these conditions, a chronically activated immune system accelerates the onset and progression of cardiovascular complications (Emanuela et al. 2012). Circulating innate immune cells, including granulocytes (Gc's) and monocytes (Mc's) normally express anchor molecules that mediate adhesion to the endothelium and migration through the vessel layers into surrounding tissues (where monocytes, for instance, may differentiate into macrophages). Some metabolic features of obesity and T2DM such as prolonged elevated postprandial glucose and lipid levels, may induce activation of both the endothelium and of these cell types, accelerating and altering movement across the intimal layer and initiating the process of formation of atherosclerotic plaques (Packard and Libby 2008). Activated Gc's and Mc's express adhesion surface molecules which facilitate their attachment to the endothelium. This process involves cell rolling, firm adhesion and diapedesis, and becomes more apparent with the progression of the initial atherosclerotic lesion (Weber and Noels 2011). Surface adhesion markers such as CD11b (Gc- and Mc-specific) and CD66b (Gc- specific) are elevated in patients with ischemic heart disease (Kassirer et al. 1999) and T2DM (van Oostrom et al., 2004b); CD14 expression was also increased in T2DM patients with cardiovascular disease (Patino et al. 2000). Other adhesion markers, such as Gc and Mc CD62L, have not been reported as chronically elevated in T2DM (van Oostrom et al., 2004b), and may actually show both an acute increase or reduction in surface expression during activation (the latter due to release form the cell surface after interaction with proinflammatory stimuli) (van Oostrom et al., 2004b).

Postprandium is a dynamic state, in which meal absorption may generate unique physiological conditions. Emerging evidence indicates that postprandial hyperglycemia, especially if occurring as frequent hyperglycemic spikes, is at least partially responsible for Gc and Mc activation and altered endothelial function (Ceriello 1998; Razmara et al. 2007) in T2D subjects, possibly predicting cardiovascular events (Cavalot et al. 2006). Similarly, following an oral sucrose load, CD11b mRNA expression increased in streptozocin-treated rats (Mochizuki et al. 2010) and the same adhesion marker significantly increased in the monocytes of both healthy and T2D human subject groups during a glucose challenge (Sampson et al. 2002).

Postprandial hyperlipidemia also displays important proinflammatory effects, contributing to the atherogenic potential of certain diets (Klop et al. 2012). Delayed clearance of lipid byproducts from the blood, for instance, has been shown in patients with coronary artery disease (Groot et al. 1991). Further, in contrast with postprandial hyperglycemia, physiologically lasting no more than 2 h after absorption of ingested carbohydrates, increased blood lipid levels may remain elevated for many hours, prolonging exposure time to this stimulus. High-fat feeding, in fact, has been shown to increase the number of leukocytes, the expression of CD11b, CD66b in neutrophils, the number of CD11b- positive neutrophils with an increase of CD62L in rats and a decrease in humans (van Oostrom et al., 2004a; Magne et al. 2010). These data parallel in vitro results in Gc's cultured in high lipid concentrations, which increased the expression of CD11b and CD66b (Wanten et al. 2000). The effects of a high-fat meal were also studied in a number of human experiments in which some level of inflammatory activation was consistently detected, albeit with enormous differences across reports (Herieka and Erridge 2014). Importantly, the overwhelming majority of studies were on healthy subjects, only a handful on obese subjects and just two on T2D patients, with no studies combining two, let alone all three, of these groups. Experimental conditions and measured variables varied wildly across studies; most commonly, only circulating inflammatory markers were measured, with variable and, at best, very moderate increases. Leukocyte surface markers were more consistently responsive, but they were only measured in a subset of studies on healthy subjects, preventing meaningful, direct comparisons (Han et al. 1999).

The proinflammatory state reflected by leukocyte surface marker expression is often paralleled by increased systemic levels of cytokines and chemokines (secreted by leukocytes themselves and by the endothelium and other tissues), whose signaling modulates the degree of inflammatory activation and directs/concentrates cells to specific locations. Among the molecules most commonly associated with these processes are TNF-α (elevated in insulin resistance and obesity in both animal and human models) (Shin et al. 2008; Kleinbongard et al. 2010); IL-6 (present within atherosclerotic lesions and systemically elevated in obesity and T2DM) (Roytblat et al. 2000; Mirza et al. 2012); MCP-1, a powerful monocyte chemoattractant (Strieter et al. 1989), (whose systemic levels were elevated in obesity subjects (Kim et al. 2006) and, in at least some reports, in T2DM) (Harsimran et al. 2009); IL-8, a potent Gc chemoattractant, (elevated in obesity and T2DM) (Esposito et al. 2003; Kim et al. 2006); and IL-1ra, a primarily anti-inflammatory molecule secreted by Mc's, macrophages, and smooth muscle cells (von der Thusen et al. 2003) (elevated in atherosclerosis, obesity and T2DM) (Meier et al. 2002; Herder et al. 2009).

Transcription of the genes of several of the inflammatory cytokines, chemokines, and adhesion markers implicated in obesity and T2D is regulated by powerful transcription factors, such as nuclear factor kappa B (NF-κB). Deriving from larger precursors, such as NF-κB-1 and -2, activated NF-κB operates through several effector subunits, including p50, p52, and p65. Sustained NF-κB activation appears to have a role in insulin-mediated glucose transport (Patel and Santani 2009) and NF-κB's p65 subunit has been implicated in the regulation of insulin sensitivity by blocking proliferator activator receptor gamma (PPR-gamma) causing insulin resistance (Ruan et al. 2003). In addition, the expression of both NF-κB p65 and p50 subunits is increased in vitro by exposure to free fatty acids (Barma et al. 2009), and in both human aortic endothelial cells and vascular smooth muscle cells by prolonged exposure (3 to 24 h) to hyperglycemia (25–55 mmol/L) (Luppi et al. 2008). An additional mechanism by which leukocyte activation is linked to atherogenesis, is the expression of specific chemokine receptors, such as the C-C motif chemokine receptor (CCR2), which facilitates the recruitment of monocytes/macrophages to visceral adipose tissue and atheromas (Sullivan et al. 2013). Increased CCR2 mRNA was reported in Mc from patients with hypercholestererolemia (Han et al. 1999), and CCR2 blockage significantly improved glycaemic parameters in T2DM patients (Di Prospero et al. 2014), and its blockage reduced the areas of atherosclerotic lesions (Okamoto et al. 2012).

The above several lines of evidence seem to clearly indicate that the inflammatory activation of innate immune cells, as reflected by cell surface marker expression and systemic cytokines levels, and stimulated by macronutrients such as glucose and lipid, play a significant role in the development and progression of atherosclerosis. This evidence, however, was fragmented across multiple studies with broadly different experimental conditions and patient characteristics. Therefore, we designed the present study to gather preliminary information on the ability of intravenously administered glucose and lipids, separately or in combination, to distinctly modulate the pattern of proinflammatory activation in innate immune cells in in healthy, obese, and diabetic patients.

## Methods

### Study subjects

Five healthy-weight, five nondiabetic obese subjects, and five T2DM patients participated in the study (subjects’ characteristics are shown in Table1). Group inclusion criteria: healthy: BMI < 30, (group avg 26 kg/m^2^); obese: BMI > 35 kg/m^2^ (group avg 39 kg/m^2^); both healthy and obese subjects had no presence or history of acute or chronic conditions, and were on no medications. T2DM patients: documented clinical diagnosis of diabetes at least 2 years prior to enrollment; history of repeated morning blood glucose level over 200 mg/dL despite treatment, currently on a regimen of antidiabetic medications. All subjects were nonsmokers; one subjects in the healthy group had a BMI of 31, due to muscular, and adipose, tissue distribution. Each subject participated in three separate visits, separated by at least 2 weeks, during which either hyperglycemia alone, hyperlipidemia alone, or both, were induced via i.v. infusions; the sequence of the three interventions was randomized. The study was approved by the University of California, Irvine Institutional Review Board (IRB), and all participating subjects signed the related informed consent documents after they had been read and explained to them by authorized study staff.

**Table 1:** Demographics of subjects including height, weight, body mass index, and vital signs

Subject Groups (males)	Age (years)	Height (cm)	Weight (kg)	BMI	Systolic Blood Pressure (mmHg)	Diastolic Blood Pressure (mmHg)	Heart Rate
Healthy (5)	30.02 ± 5.38	172.80 ± 4.30	76.38 ± 3.92	25.76 ± 2.02	121.6 ± 3.25	71.8 ± 3.38	69.8 ± 3.91
Obese (5)	29.79 ± 4.60	180.57 ± 3.30	144.68 ± 9.68***	44.29 ± 2.30***	137.4 ± 6.87	85 ± 3.69	64.2 ± 4.66
T2DM (5)	46.28 ± 3.94*	173.60 ± 2.06	91.92 ± 5.90	30.45 ± 1.60	133.6 ± 14.1	83.8 ± 7.01	70.8 ± 4.51

The data were analyzed with One-Way Analysis of Variance (ANOVA) ^*^^*^^*^P < 0.001 Obese versus Healthy, ^*^P < 0.05 T2DM versus Healthy. Data are mean ± SE.

### Study protocol

**Figure 1:**
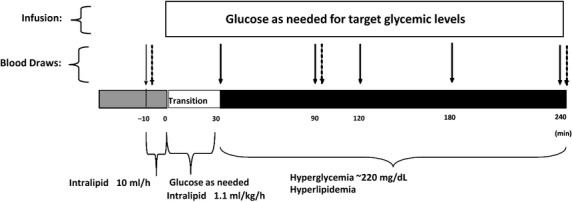
Schematic representation of glucose, lipids, and glucose + lipids infusion study design. Blood samples for cell surface markers and systemic proteins were taken at baseline and timepoints 30, 90, 120, 180, and 240 min (full arrows) and for gene expression studies at baseline, 90, and 240 min (dashed arrows).

Study participants were fasting overnight before each procedure but were allowed to drink water ad libitum. Subjects were schedule to report at the University of California, Irvine Institute for Clinical and Translational Science (UCI ICTS) at 7:30 am on study day (actual admission time was never more than 30 min off scheduled time). Following the subjects’ arrival, a physical examination was conducted to confirm that vital signs (blood pressure, heart rate) were within normal limits (Table1). Intravenous catheters were inserted in the subjects’ antecubital veins on both arms for glucose/lipid infusion and blood drawings. Baseline samples were then drawn, and the infusion of glucose (20% dextrose solution) and/or Intralipid (an emulsion of 20% soy bean oil, 1.2% egg yolk phospholipids, and 2.25% glycerin and 76.55% water. This solution contains fatty acids including 44–62% linoleic, 19–30% oleic, 7–14% palmitic, 4–11% linolenic, and 1.4–5.5% stearic) were started. Blood samples for inflammatory cell surface markers and for systemic cytokines and chemokines were drawn at baseline and at timepoints 30, 90, 120, 180, and 240 min; (30, 90, and 240 min for RT-PCR measurements) (Fig.1). In studies requiring hyperglycemia, plasma glucose was increased to 220 mg/dL over 30 min, and clamped at this level for the remaining 210 min of the study (Fig.1). Plasma glucose was measured every 5–10 min during the study with a Beckham Glucose Analyzer (Fullerton, CA), and the glucose infusion rate adjusted accordingly to maintain target glycemia. In studies requiring hyperlipidemia, the Intralipid infusion was started with a slow (10 mL/h) infusion for 10 min, in order to identify any possible allergic reaction to the nonlipid component of the infusate (tachycardia, altered breathing, skin reactions, etc.), and subsequently increased to 1.1 mL/kg/h (Fig.1).

### Flow cytometry measurements

For surface marker flow cytometry measurements, a 100 μL whole blood sample from these tubes was mixed on a Vortex mixer (Scientific Industries Inc, Bohemia, NY), and incubated in the dark for 15 min with undiluted fluorescent-conjugated monoclonal antibodies specific to CD11b-phycoerythrin (PE) (Becton Dickinson/Pharmigen San Diego, CA), CD14-fluorescein isothiocyanate (FITC), (AlexaFluor 647) (Biolegend San Diego, CA), CD16-PE (Biolegend San Diego, CA), CD62L-FITC (Becton Dickinson/Pharmigen San Diego, CA), and CD66b-FITC (Becton Dickinson/Pharmigen San Diego, CA). CD66b is a specific marker for granulocytes; CD11b is specific for granulocytes and monocytes; CD14 and CD16 simultaneous expression is specific to monocytes, with the degree of CD16 expression reflecting cell activation; CD62L is expressed both on granulocytes and monocytes, is involved endothelial adhesion, and is shed from the cell surface when other proinflammatory markers, such as CD11b, are activated.

Red blood cells were then lysed with 2 mL 1× FACS lysing solution (Becton Dickinson, San Jose, CA) and the samples were incubated for 15 min in dark. The samples were centrifuged at 600 g for 10 min and washed in 2 ml 1× wash buffer containing 1% fetal calf serum and 0.02% sodium azide in 1× phosphate-buffered saline (PBS) without calcium and magnesium. Each sample was centrifuged at 600 g for 10 min, the supernatant was removed, and the pellet was resuspended in 500 μL 2% formaldehyde in PBS.

An Accuri C6 (BD Accuri Cytometers Ann Arbor, MI) flow cytometer was used for data acquisition and analysis. Each event was recorded in a forward-side scatter plot in which lymphocytes, monocytes, and granulocytes were located according to their low, medium, or high forward scattering and the data were reported as mean fluorescence intensity (MFI) normalized to three thousand monocytes.

### Enzyme-linked immunosorbent assay (ELISA) measurements

Blood samples collected in EDTA tubes (Fig.1) were centrifuged at 600 g for 10 min, the plasma was removed, and placed into −80°C until assay day. On assay day, high sensitivity TNF-α (R&D System Minneapolis, MN), high sensitivity IL-6 (R&D System Minneapolis), high sensitivity IL-8 (R&D System Minneapolis), IL-1ra (R&D System Minneapolis), MCP-1 (R&D System Minneapolis) ELISA assay kits were used to measure cytokine content, according to the manufacturer's instructions. In addition, colorimetric assays detecting free fatty acids (ZenBio Research Triangle Park, NC), and triglycerides (BioAssay Systems Hayward, CA) were also perfomed.

### Quantitative Real-Time PCR Studies (qRT-PCR) measurements

#### Obtaining Peripheral Blood Mononuclear Cells (PBMC's)

Part of the blood samples (8 mL) drawn at baseline and at timepoints 90 and 240 min into vacutainer tubes (tigertop) (Fig.1) was centrifuged at 1300 g for 20 min; PBMC's were then removed and placed into 15 mL tubes. Samples were washed with PBS and centrifuged at 600 g for 15 min. The PBS was removed, the pellet was resuspended in 1 mL Trizol, transferred to smaller tubes, and were stored at −80°C.

#### Isolation of total RNA

The samples were thawed on ice; 0.2 mL chloroform was added, shaken for 15 s and incubated on RT for 5 min. Each tube was centrifuged on 15,000 g for at 4°C for 15 min and the aqueous phase was removed and placed into fresh tubes. To the aqueous phase, 0.5 mL isopropanol was added; the contents were mixed and incubated at RT for 10 min. The samples were centrifuged at 10,000 g for 10 min and the pellet was washed in 1 mL 80% ethanol. The pellet was resuspended and the samples were centrifuged at 10,000 g for 5 min. The supernatant was removed and the ethanol wash and centrifuging step was repeated as above. The supernatant was removed and the pellets were air-dried. The dry RNA pellets were resuspended in 30 μL RNAse-free water and total RNA content was measured by spectrophotometry.

#### cDNA synthesis

RNA content was normalized to 1 μg/mL concentration and diluted to 10 μL samples with RNAse-free water. Mastermix (10 μL) (Applied BioSystems Grand Island, NY) containing 2 μL 10× RT buffer, 0.8 μL 25× (100 mmol/L) dNTP mix, 2 μL 10 × RT random primers, 1 μL Multiscribe Reverse Transcriptase, 4.2 μL nuclease-free water. The samples were placed into the thermocycler (25°C 10 min, 37°C 60 min, 37°C 60 min, 80°C 5 min), diluted 1:5 with RNAse-free water and placed into −20°C.

#### qRT–PCR reaction

NFκB1, CCR2 primers (Life Technologies Grand Island, NY), Universal Mastermix II (Applied BioSystems Grand Island, NY), and samples have been prepared placing them into 96-well plates. Actin-β was used as endogenous control. At each well, 5 μL Universal Mastermix II, 1 μL specific primer, and 1 μL sample were placed. The 96-well plate was placed into a 7900HT PCR system (Life Technologies, Grand Island, NY) and 40 cycles completed (95°C–10 min, 95°C–15 s, 60°C–60 s).

### Statistics

Data are reported as group means and standard error of the mean (SEM). Across-group differences were analyzed with paired t-tests, Two-Way ANOVA, and Two-Way Repeated ANOVA followed by Bonferroni Correction tests. Statistical significance was defined as *P* < 0.05. Graphpad Prism 4 software (GraphPad Software Inc. San Diego, CA) was used for all statistical analyses.

## Results

### Cell surface marker expression

**Figure 2:**
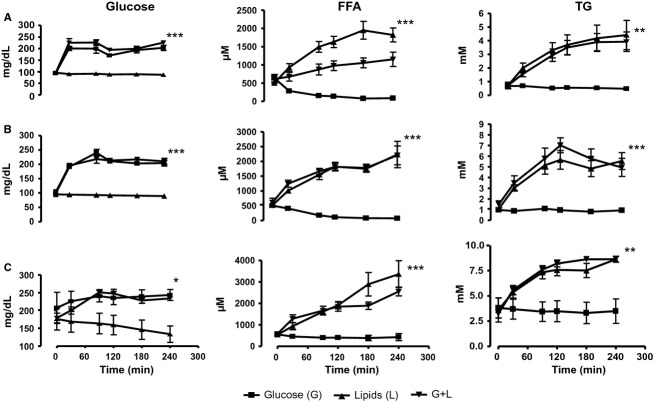
Change in concentration of glucose, free fatty acids (FFA), and triglycerides in (A) healthy, (B) obese, and (C) T2DM subjects during glucose, lipid, and glucose + lipid infusions areas under the curves were analyzed with One-Way Analysis of Variance (ANOVA) and Dunnett's Multiple Comparison Tests. (***P < 0.001, **P < 0.01, *P < 0.05)

During all studies, target values of hyperglycemia and hyperlipidemia were reached consistently in all groups (Fig.2); as expected, in the healthy group, during combined lipid and glucose infusion, FFA were lower than during lipid infusion alone, due to greater antilipolytic effect related to the greater insulin sensitivity of these subjects.

Significant leukocytosis occurred in all three groups during infusion of lipids only (Table2); this increase was smaller and nonsignificant during combined infusion of lipids and glucose, while, during infusion of glucose only, total leukocyte counts displayed a modest reduction.

**Table 2:** Complete Blood Counts (CBC) of subjects obtained measured by our clinical laboratory at baseline and end of the studies

	WBC 0 min	WBC 240 min	Neutrophil 0 min	Neutrophil 240 min	Lymphocyte 0 min	Lymphocyte 240 min	Monocyte 0 min	Monocyte 240 min
Glucose								
Healthy	4.7 ± 0.33	4.0 ± 0.25*	2.4 ± 0.33	2.1 ± 0.38	1.6 ± 0.12	1.6 ± 0.24	0.5 ± 0.12	0.3 ± 0.04
Obese	7.3 ± 0.54	6.5 ± 0.64**	4.6 ± 0.78	4.2 ± 0.52	2.0 ± 0.47	1.7 ± 0.21	0.5 ± 0.13	0.5 ± 0.10
T2DM	5.1 ± 0.38	4.9 ± 0.33	3.1 ± 0.22	2.9 ± 0.27	1.4 ± 0.14	1.5 ± 0.14	0.4 ± 0.08	0.3 ± 0.08
Lipids								
Healthy	4.5 ± 0.45	6.2 ± 0.76*	2.2 ± 0.52	3.7 ± 0.48*	1.6 ± 0.18	2.1 ± 0.47	0.4 ± 0.00	0.3 ± 0.04*
Obese	6.8 ± 0.43	7.8 ± 0.81*	4.0 ± 0.60	4.8 ± 0.92	2.0 ± 0.32	2.2 ± 0.41	0.4 ± 0.04	0.3 ± 0.09
T2DM	6.1 ± 0.29	6.8 ± 0.40*	3.7 ± 0.29	4.1 ± 0.31	1.8 ± 0.12	2.0 ± 0.19	0.4 ± 0.10	0.3 ± 0.10
G + L								
Healthy	4.6 ± 0.41	4.7 ± 0.24	2.6 ± 0.39	2.4 ± 0.27	1.3 ± 0.09	1.7 ± 0.25	0.4 ± 0.07	0.3 ± 0.08
Obese	6.8 ± 0.35	7.7 ± 0.73	4.0 ± 0.46	5.0 ± 0.65	2.1 ± 0.29	1.9 ± 0.08	0.4 ± 0.06	0.4 ± 0.15
T2DM	5.5 ± 0.38	5.8 ± 0.35	3.4 ± 0.29	3.4 ± 0.28	1.5 ± 0.10	1.9 ± 0.18*	0.4 ± 0.07	0.3 ± 0.05

The data were analyzed with Two-Tailed Paired *t*-tests (^*^^*^P < 0.01, ^*^P < 0.05). Data are mean ± SE.

At baseline, some degree of variability was observed across groups in absolute levels of surface marker expression (Table3); in general, values tended to be higher the obese group than in the other two, although significantly so only for Mc CD 62L (MFI, Obese, 15,361 ± 1002, Healthy, 11,305 ± 823, T2D 12,630 ± 512, *P* < 0.05).

**Table 3:** Baseline surface expression of CD11b, CD62L, and CD66b in Gc in addition to CD11b and CD62 in Mc of healthy, obese, and T2D subjects during glucose, lipids, and glucose + lipids infusions

Surface Marker-Group	Healthy	Obese	Type II Diabetic
CD11b (Gc)	19,798 ± 1685	24,072 ± 1960	20,470 ± 2487
CD62L (Gc)	14,228 ± 1081	17,001 ± 1105	16,843 ± 766
CD66b (Gc)	14,740 ± 705	11,918 ± 585	15,095 ± 1022
CD11b (Mc)	11,773 ± 766	12,533 ± 840	14,103 ± 1386
CD62L (Mc)	11,305 ± 823	15,361 ± 1002*	12,630 ± 512

The data were analyzed with One-Way Analysis of Variance (ANOVA) and Dunnett's Multiple Comparison tests (^*^P < 0.05). Data are mean ± SE.

**Figure 3:**
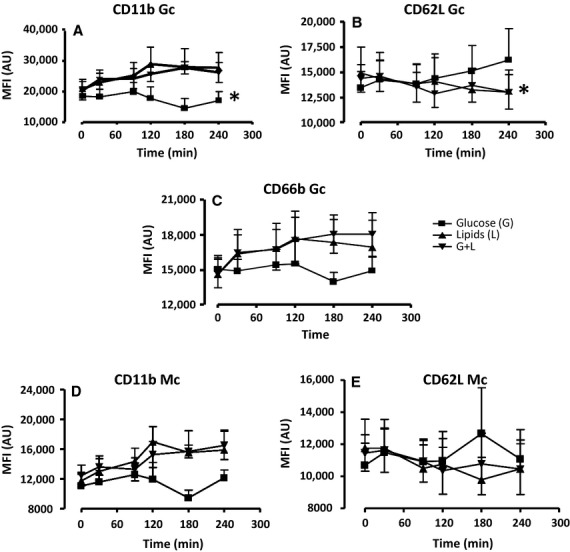
The effects of a 240-min glucose (G), lipids (L), and a glucose/lipids (G + L) combination clamp on (A) CD11b, (B) CD62, and (C) CD66b of Gc and (D) CD11b and (E) CD62L of Mc in healthy subjects (*n* = 5). The data were analyzed with Two-Way Repeated Analysis of Variance (ANOVA) and Bonferroni Corrections. The * represents overall significance (*P < 0.05).

In healthy subjects, lipids and glucose + lipids infusions resulted in elevated expression of CD11b in Gc (∼40% increase at 240 min as compared to baseline); conversely, infusion of glucose alone induced no significant change in this variable over baseline (*P* < 0.05 vs. other treatments, Fig.3A). In addition, Gc CD62L expression was reduced below baseline during lipid and glucose + lipid infusions, but not during glucose-only infusion (*P* < 0.05 vs. other treatments, Fig.3B). Gc CD66b expression, similar to CD11b, increased over baseline during lipid and glucose + lipids infusions but did not change with glucose-only treatment; in this case, however, statistical significance across treatments was not reached (Fig.3C). Mc CD11b expression in healthy subjects had a similar pattern as in Gc, displaying a 20% increase compared to baseline during lipid and glucose + lipids infusions albeit again without reaching statistical significance across treatments (Fig.3D). Mc CD62L expression only slightly decreased over time compared to baseline without significance change across the treatment groups (Fig.3E).

**Figure 4:**
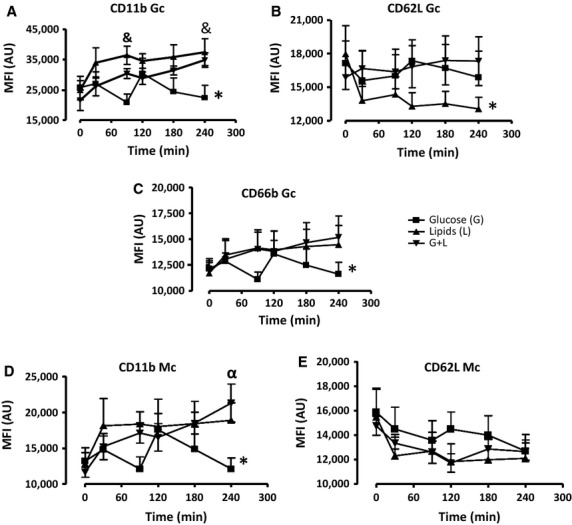
Expression of activation surface markers (A) CD11b (B) CD62L (C) CD66b of Gc and (D) CD11b and (E) CD62L of Mc in obese subjects (*n* = 5) during a 240-min glucose (G), lipids (L) and glucose + lipids (G + L) combination clamp studies. The data were analyzed with Two-Way Repeated Analysis of Variance (ANOVA) and Bonferroni Corrections. The * represents overall significance (*P < 0.05). (^&^*P* < 0.05) glucose versus lipids treatment.

In obese subjects, Gc CD11b and CD66b expression increased significantly during lipid and glucose + lipids infusions, with highest levels roughly doubling baseline values for CD11b, and showing a ∼30% increase for CD66b (Fig.4C). Lipids-only infusion showed the highest level of activation reaching significance at the 90 and 240 min timepoints, while glucose-only infusion resulted in a see-saw pattern of activation, and the lowest overall CD11b expression values compared to other treatments (Fig.4A). Gc CD62L expression rapidly and significantly decreased during the time of lipid-only infusion, compared to glucose-only and glucose + lipids studies, which both remained at values 25–30% higher values at 240 min (*P* < 0.05). During lipid infusion Gc CD62L expression decreased ∼30% compared to baseline (Fig.4B). Similar to Gc, Mc expressions of CD11b was also significantly increased by ∼40% with lipids-only and glucose + lipids as compared to glucose-only treatment, displaying the greatest difference at the 240 min timepoint. In addition, a ∼40% increase was also observed during the same treatment compared to baseline (Fig.4D). CD62L showed a strong tendency to decrease with all treatments compared to baseline and while this was more pronounced during the early time points with lipids and glucose + lipids infusions statistically significant differences between groups were not observed and by 240 min all groups had again comparable values (Fig.4E).

**Figure 5:**
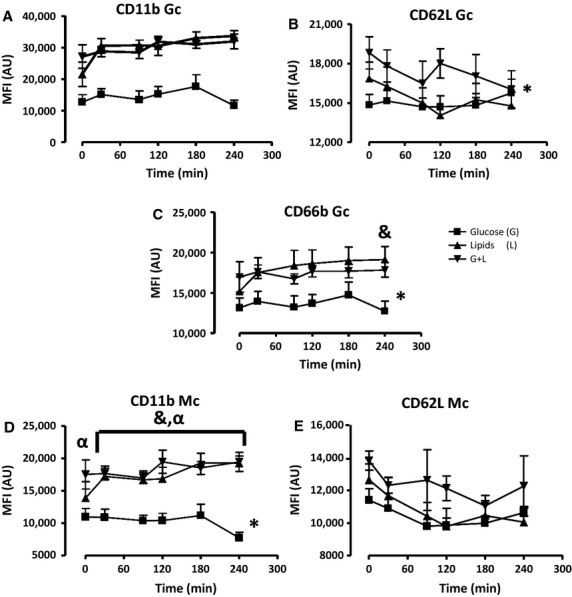
The expression of (A) CD11b (B) CD62L (C) CD66b of Gc and (D) CD11b and (E) CD62L of Mc in patients with T2DM (*n* = 5) during a 240-min glucose (G), lipids (L), and glucose + lipids (G + L) combination clamp. The data were analyzed with Two-Way Repeated Analysis of Variance (ANOVA) and Bonferroni Corrections. The * represents overall significance (*P < 0.05). (^&^*P* < 0.05) glucose versus lipids treatment, (^α^*P* < 0.05) glucose versus G + L.

In T2DM, baseline values displayed a much greater degree of baseline variability than in the other two groups, with lowest values measured with glucose-only studies, and highest values with glucose + lipid studies. Glucose-only infusion had little effect on all measured variables in GC compared to baseline but lipid-only infusion, on the other hand, resulted in a sharp activation of Gc CD11b and CD66b from 0–30 min. During the glucose + lipids treatments, CD11b and CD66b (*P* < 0.05) were markedly greater compared to glucose-only study, (roughly at the same level as in lipid-only studies after the first 30 min) with the greatest difference observed at 240 min (Fig.5A and C). Gc CD62L showed a marked, parallel decrease with both lipids-only and glucose + lipids, but was unchanged during glucose-only infusions compared to baseline; however, did not show significant differences across treatment groups (Fig.5b).

The same pattern of baseline differences across conditions was observed with Mc. Also, similar to Gc, Mc expression of CD11b increased rapidly and remained elevated during lipids-only infusions, with values similar to glucose + lipids in which, however, values again did not increase over baseline. Glucose-only studies showed no baseline effects on these markers (Fig.5D). CD62L expression in Mc decreased similarly with all treatments during the first half of the study compared to baseline and did not show any significant differences across groups (Fig.5E). Additional surface markers including CD14 and CD16 did not show significant differences during glucose, lipids, and glucose + lipids infusions across groups (data not shown).

### Circulating protein markers

**Figure 6:**
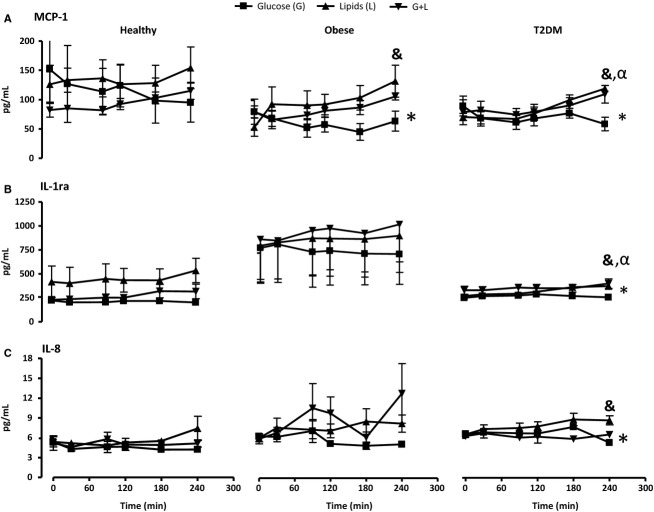
The effects of glucose, lipids, and glucose + lipids infusions on the plasma. (A) MCP-1 (B) IL-1ra and (C) IL-8 of healthy, obese and T2DM subjects. The data were analyzed with Two-Way Analysis of Variance (ANOVA) (for healthy) and Two-Way Repeated Analysis of Variance (ANOVA) (for obese and T2DM) and Bonferroni Corrections. The * represents overall significance (*P < 0.05). (^&^*P* < 0.05) glucose versus lipids treatment, (^α^*P* < 0.05) glucose versus G + L.

In healthy subjects, the circulating chemokine MCP-1 (Fig.6A) and other measured cytokines did not show any significant change from baseline during study infusions, nor displayed a consistent trend in either treatment during the 240 min sessions.

Conversely, in obese subjects MCP-1 increased significantly during lipids-only and lipids + glucose (Fig.6A). A similar trend, albeit of smaller magnitude, was observed for IL-8 at most timepoints, more pronounced during the glucose + lipids, although it did not reach statistical significance (Fig.6C). IL-1ra changes were limited to a moderate increase during glucose + lipids infusion experiments. Interestingly, baseline IL-1ra levels were higher in obese subjects than in healthy controls (Fig.6B).

Similar to obese subjects, in patients with T2DM, lipid and glucose + lipids infusions resulted in a significant increase in circulating MCP-1 levels compared to glucose infusion alone at 240 min (Fig.6A). Similarly, IL1ra level in these patients during lipid and glucose + lipids infusions was approximately 40% greater at 240 min compared to glucose-only infusions (Fig.6B). In addition, lipid-only treatment (but not glucose + lipids) resulted in a significant increase in IL-8 release (Fig.6C). Baseline values for patients with T2DM for MCP-1 and IL-8 corresponded approximately those of the obese subjects, while IL-1ra was similar to healthy volunteers’ plasma TNF-α and IL-6 did not show significant changes in either group (data not shown).

### PBMC gene expression

NFκB expression of PBMC's did not show any statistically significant change during experimental procedures in any of the three subject groups (Table4). The baseline expression of NFκB was somewhat variable, with higher levels in lipid and glucose + lipids studies as compared to glucose-only; levels of expression, however, remained similar to baseline and stayed higher during the 240 min.

**Table 4:** NFκB and CCR-2 gene expressions of PBMC's during glucose, lipids, and glucose + lipids infusions in healthy (*n* = 5), obese (*n* = 5), and T2DM (*n* = 5) subjects

Time (min)	Healthy			Obese			T2DM		
	Glucose	Lipids	G + L	Glucose	Lipids	G + L	Glucose	Lipids	G + L
NFKB									
0	0.62 ± 0.1	1.44 ± 1.0	0.88 ± 0.5	0.25 ± 0.1	0.49 ± 0.2	0.39 ± 0.1	0.06 ± 0.0	0.37 ± 0.1	0.47 ± 0.2
90	1.12 ± 0.4	0.61 ± 0.4	0.57 ± 0.2	0.27 ± 0.1	0.36 ± 0.1	0.32 ± 0.1	0.07 ± 0.0	0.47 ± 0.2	0.69 ± 0.5
240	1.08 ± 0.6	0.34 ± 0.1	1.04 ± 0.5	0.21 ± 0.1	0.41 ± 0.1	0.24 ± 0.0	0.03 ± 0.0	0.48 ± 0.2	1.10 ± 0.8
CCR2									
0	0.67 ± 0.1	0.75 ± 0.2	1.05 ± 0.2	0.38 ± 0.1	0.73 ± 0.3	1.10 ± 0.3	0.13 ± 0.0	0.45 ± 0.1	0.51 ± 0.1
90	1.02 ± 0.3	0.48 ± 0.1	0.50 ± 0.1	0.59 ± 0.2	0.42 ± 0.1	1.50 ± 1.3	0.14 ± 0.0	0.53 ± 0.3	0.48 ± 0.2
240	2.43 ± 1.9	0.46 ± 0.2	0.44 ± 0.1	0.38 ± 0.1	0.44 ± 0.1	0.72 ± 0.5	0.10 ± 0.0	0.32 ± 0.1	0.29 ± 0.1

Data are mean ± SE.

Similarly the PBMC gene expression of the MCP-1 receptor, CCR-2, in healthy, obese, and T2DM subjects, did not show significant differences during glucose, lipid, and glucose + lipid infusions. In addition to the NFκB data, CCR2 gene expression at baseline also shows elevated values in lipid and glucose + lipid infusions, and the gene expression for these treatments was higher throughout the 240 min course (Table4).

## Discussion

In this series of experiments, we evaluated the effects of acute hyperglycemia, hyperlipidemia, and a combination of the two on activation of Gc and Mc surface markers and concentrations of circulating inflammatory cytokines in healthy, obese, and T2DM subjects. Although a few studies have been published reporting the use of glucose and lipid clamp techniques to induce controlled systemic changes of these substances in cats (Zini et al. 2010) and humans (Esposito et al. 2002), the literature is scarce in data generated by infusing multiple substrates in different populations of human subjects. Overall, our data, although obtained from small groups of subjects, showed a more pronounced effect on the expression of adhesion surface marker when lipids were infused. Conversely, in our experimental setting glucose displayed little or no acute effect, either alone or in combination with lipids. It should be noted that the levels of the induced increases in plasma glucose and lipids, although considerable in magnitude, are very relevant to everyday life in patients with dysmetabolic conditions. Glycemic levels similar to those induced in the study, although supraphysiological in healthy subjects, are unfortunately very common after a meal in diabetic patients, even in those with average to good glycemic control. The induced levels of plasma triglycerides and FFAs, that is, about a fourfold increase over baseline; while unlikely to be observed in real life in response to a single fatty meal ingestion, are not unusual in severe chronic hyperlipidemia. In fact, even in our study, the highest TG levels measured in the healthy subjects during lipid infusion were barely greater than the baseline plasma value reported in the T2DM group.

The significance of CD11b in atherosclerosis has been shown in multiple models although human studies tend to be inconsistent. Transgenic mice in which CD11b-carrying Mc were depleted, for instance, displayed decreased size and necrotic core development in atherosclerotic plaques (Stoneman et al. 2007). Although in one study in morbidly obese subjects, CD11b was not expressed at different levels than in healthy controls (Cottam et al. 2002) and Mastej and colleagues did not observe a significant increase of CD11b in neutrophils of T2D subjects who did not have vascular complications (Mastej and Adamiec 2009), another study by Boschmann et al. showed that obese hypertensive subjects with high Mc CD11b had increased concentrations of glucose in the adipose tissue following a glucose tolerance test compared to controls (Boschmann et al. 2005). Expression of Gc CD66b, on the other hand, was observed to be increased in healthy volunteers following ingestion of a high-fat meal (van Oostrom et al., 2004a), and in T2D subjects who do not have coronary heart disease (van Oostrom et al., 2004b); further, CD66b-positive neutrophils are consistently found in increased numbers within rupture-prone atherosclerotic lesions (Ionita et al. 2010). CD62L also helps the adhesion of leukocytes to the endothelium, and its absence has been observed to cause the impairment of recruiting leukocyte to the injury site (Tedder et al. 1995). This marker, however, is involved in early weak binding and is subsequently shed as cell activation proceeds further, explaining the decrease on cell surface that was observed in our study during prolonged activation (van Oostrom et al., 2004a).

Determining the mechanism by which cell surface markers expression increased in Gc's and Mc's was beyond the scope of our study. We believe, however, that this may have occurred in one of three possible ways: (1) by mobilization of already activated cells from the marginated pool, with the lungs as the main single repository; (2) by modification of maturational processes in the bone marrow; (3) by activation of mature cells already present in the bloodstream. We believe all three mechanisms are possible in our experimental setting. Mobilization of leukocytes, and especially neutrophils, indeed occurred to a variables degree during our experiments (Table2). Whether these mobilized cells had a different level of proinflammatory action, however, remains unclear. Increased bone marrow production of both Gc's and Mc's in response to both hyperglycemia and hyperlipidemia, as well as increased inflammatory activation of released cells (Drechsler et al. 2010), has also been documented in animal models. These mechanisms, however, may be more relevant for chronic exposures to elevated levels of these metabolites. Acute activation of mature circulating cells has also been demonstrated by culture studies of leukocytes exposed to increasing concentrations of glucose and lipids (Drechsler et al. 2010). It should also be noted that to initiate the atherogenic process, in addition to immune cell activation, target tissues, such as the endothelium, need to become “receptive” via expression, for instance, of adhesion molecules. While endothelial activation was not directly measured in our study, it is well documented, (Dart and Chin-Dusting 1999) that lipid exposure per se, even at levels lower than those generated in our experiments, can indeed induce these endothelial changes and facilitate leukocyte adhesion. It is, therefore, reasonable to infer that endothelial activation occurred simultaneously with leukocyte activation in our experimental setting**.** An additional reason of concern in data interpretation is also the well accepted heterogeneity that exists within and among healthy individuals in immune cell surface marker patters, especially concerning monocytes. Our experimental protocol, however, was designed to account for this problem. In our healthy and obese groups, baseline values of measured surface markers were remarkably stable across conditions (albeit admittedly less so in the T2D group, for understandable metabolic instability); more importantly, each subject being his own control, acute changes in cell surface markers are relative to baseline values, accounting for baseline differences. Determining the relative contribution of the multiple mechanisms activating immune cells in vivo, and directly assessing the degree of simultaneous activation of endothelial cells, can constitute the intriguing rationale for more in-depth future studies.

The reported changes in cell surface marker activation were paralleled, in obese and T2DM subjects, by small but measurable increases in systemic cytokine/chemokine levels (MCP-1 in obese. and MCP-1, IL-1ra, and IL-8 in T2D), again during lipids-only and glucose + lipids infusions. Conversely, in healthy volunteers no significant changes were observed. MCP-1 is released by circulating Mc, by macrophage embedded within the adipose tissue (Sartipy and Loskutoff 2003), and, possibly in the largest amounts, by the endothelium (Ohman et al. 2010). Human umbilical vein endothelial cells (HUVEC), for instance, displayed large increases in MCP-1 mRNA expression in vitro, but only after prolonged exposure to very high glucose concentrations (25–35 mmol/L) (Takaishi et al. 2003). This may possibly explain why our relatively short exposure to lower glycemic levels (appr.14 mmol/L) may not have been able to reproduce a similar increase.

The anti-inflammatory cytokine IL-1ra,which counteracts the proinflammatory effects of IL-1α and IL-1β, is released by the adipose tissue, and was reported to be increased in obesity and inflammation (Juge-Aubry et al. 2003); consistent with these prior observations, our obese group displayed consistently higher IL1-ra than the other two groups. Our experimental procedures, however, did not induce any acute changes in IL-1ra in any of the groups, suggesting that chronic dyslipidemia may be needed for a systemic increase in this cytokine; this hypothesis is also supported by the prior observation that acute lipid infusion in healthy subjects did not result in increased systemic IL-1ra expression (Nowotny et al. 2013). The neutrophil chemoattractant IL-8 is also released from the adipose tissue and tends to increase with weight gain (Fain 2006); however neither IL-8 gene expression nor protein secretion increased significantly on dermal microvascular endothelial cells during a 24-h 30 mmol/L glucose exposure (Jain et al. 2011), suggesting that a stronger/longer stimulus may be needed.

To our knowledge, mRNA expression of key regulators of innate immune system activations has not been investigated simultaneously in vivo in multiple dysmetabolic populations. In our study measured by RT-PCR, in PBMC's, the mRNA expression of the transcription factor NFκB, which controls the transcription of multiple inflammatory proteins including MCP-1, TNF-α (Quan et al. 2011), IL-8, and IL-6 (Hoffmann et al. 2002) (Libby 2002), and of the chemokine receptor CCR2, involved in the recruitment of monocytes/macrophages to visceral adipose tissue and atherosclerotic lesions. In our study, acute changes in mRNA expression of these molecules were not observed in PBMC's, suggesting that either stronger or more prolonged stimuli may be needed to cause measurable effects. Interestingly, however, differences in the NFκB and CCR2 mRNA were present at baseline, within the same group of subjects, across experimental days. This finding was especially pronounced in the T2D group, paralleling baseline differences in Gc and Mc surface marker expression (Table3). This may indicate that even in our experimental setting mRNA expression of these factors may still reflect overall inflammatory status, while detection of acute changes may require substantially more prolonged observation times.

We openly acknowledge that our study has a series of limitations. While a total of 45 metabolic studies was included in the analysis, in fact, group size (*n* = 5) was certainly small. Further, due to the high cost and relative discomfort for subjects, a “pure” control group was not included, with infusion of saline only; available reports, however, indicate it is extremely unlikely that time alone could have any tangible activating effect of surface markers expression (El-Raggal et al. 2004). Also, the condition eliciting the greater leukocyte responses, infusion of lipids, was achieved utilizing a commercial lipid emulsion, which also includes small amounts of glycerin and egg yolk phospholipids. While it is unlikely that these compounds have contributed to the observed increased expression of cell surface markers, this effect cannot be completely excluded unless they are infused separately, a procedure that could not be included in the current study protocol.

The lipid composition of the infused emulsion, on the other hand, is based on FFA distribution in soy products, and therefore reflects only one of many possible combinations of FFA in common nutrients and dietary regimens, which vary extensively across individuals. The five FFAs present in the emulsion, however, (Linoleic, Linolenic, Oleic, Palmitic, and Stearic) represent 90–97% of total FFAs present in human blood, the main difference consisting of a substantially greater % of linoleate and lower % of palmitate. This particular lipid mixture, therefore, is not unlikely to be ingested in an average person's diet. Another study limitation may be seen as the fact that we only investigated two possible molecular pathways of cell activation, that is, the expression of NFκB and CCR2, while relevant information could have been gathered by studying, for instance, the nuclear localization of NFκB itself, or the levels of activation/degradation of IκB. Important information could have also been gathered via coculture studies of monocytes and endothelial cells exposed to plasma drawn during glucose and lipid infusions. While these additional experiments could not be performed at the present time, they certainly represent a logical possible expansion of this study in the future.

It should be noted that, as the mean age of members of the T2D group was higher than the other two, results could have been affected by this difference. It has indeed been reported that systemic inflammatory status increases with age (Franceschi and Campisi 2014), but several studies also reported little or no difference in the level of expression of individual leukocytes surface markers, including CD11b, between elderly and young subjects (Glynn et al. 1999; Noble et al. 1999). Further, in our study, for five of the reported surface markers, the highest values at baseline were actually in the obese group, which had the same age as the controls, and only one marker, Mc CD11b, had clearly higher values in T2D than in the other two groups (and even in this case, the difference was <20%). We, therefore, believe that age had a minimal, if any, effect on our main observations.

Current standard-of-care for obesity and T2DM (Pahan 2006) includes lipid-lowering medications (statins, peroxisomal β pathway stimulators, intestinal cholesterol absorption inhibitors), and, before insulin injections become necessary, oral hypoglycemic agents (insulin sensitizers and secretagogues, delaying agents of gut carbohydrates absorption). The therapeutic use of these molecules is not only often relatively ineffective but also exposes patients to serious side effects such as the worsening of diabetes (Marcus et al. 2013), rhabdomyolysis (Amend et al. 2011), severe headaches (Domagala et al. 2003), liver failure, etc. Fully clarifying the biochemical details of how exposure to glucose and lipids activates innate immune cells could considerably improve management and prevention of complications in obesity and diabetes. Quantifying the presence and level of cell activation may allow, for instance, to select and monitor the effect of tailored dietary guidelines, and/or to develop more focused anti-inflammatory agents, targeting specifically the involved pathways and mediators, thereby potentially reducing side effects. Our small but complex study is one step in the direction of unveiling possible future therapeutic targets along the biochemical pathways linking immune cell activation and progression of atherogenic processes.
